# Mixed-solvent liquid exfoliated MoS_2_ NPs as peroxidase mimetics for colorimetric detection of H_2_O_2_ and glucose†

**DOI:** 10.1039/c7ra12584d

**Published:** 2018-02-15

**Authors:** Yuehua Zhao, Yu Huang, Jiangling Wu, Xiaoli Zhan, Yuanyang Xie, Dongyun Tang, Haiyan Cao, Wen Yun

**Affiliations:** Chongqing Institute of Green and Intelligent Technology, Chinese Academy of Sciences Chongqing 400714 China huangyu@cigit.ac.cn; University of Chinese Academy of Science Beijing 100049 China; Department of Clinical Laboratory, University-Town Hospital of Chongqing Medical University Chongqing 401331 China; Chongqing Traditional Chinese Medicine Hospital Chongqing 400021 China; School of Chemistry and Chemical Engineering, Yangtze Normal University 408100 Chongqing China 513923170@qq.com; Chongqing Key Laboratory of Catalysis and Functional Organic Molecules, College of Environment and Resources, Chongqing Technology and Business University Chongqing 400067 China 44863542@qq.com

## Abstract

Ultra-small molybdenum disulfide nanoparticles (MoS_2_ NPs) were prepared by a facile liquid exfoliation method with ethanol/water as the solvent. The produced MoS_2_ NPs were of high purity due to the easily removable ethanol/water solution. The prepared MoS_2_ NPs exhibited an intrinsic peroxidase-like activity in analogy to that of horseradish peroxidase (HRP). A custom-made spectrometer was employed to investigate the peroxidase-like activity of MoS_2_ NPs in the presence of H_2_O_2_ and glucose. The change in absorption detected from MoS_2_ NPs is proportional to the amount of target. The calibration curve of H_2_O_2_ and glucose shows a good relationship between the concentration of target and the change in the absorption of MoS_2_ NPs. The limit of detection of H_2_O_2_ and glucose achieved by this method could approach 1.25 μM and 7 μM respectively. This method has been applied for the detection of glucose in serum from humans. Therefore, these produced MoS_2_ NPs offer an alternative high-efficiency and economic way to detect diabetes.

## Abstract 2

Ultra-small molybdenum disulfide nanoparticles (MoS_2_ NPs) prepared by a facile liquid exfoliation method is capable of detecting the presence of H_2_O_2_ and glucose. This novel colorimetric method offers an alternative way to detect diabetes.

## Graphical abstract

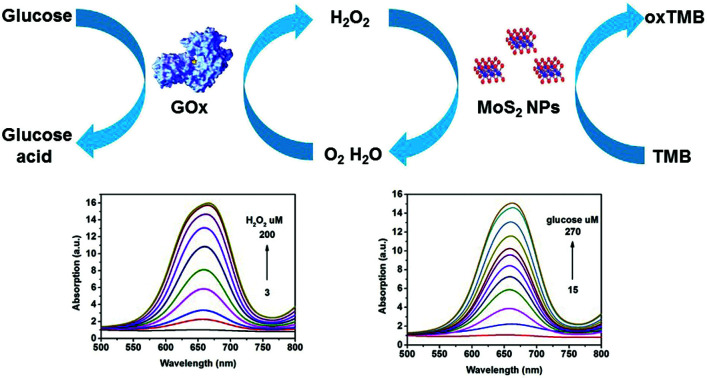

## Introduction

The natural enzyme, horseradish peroxidase (HRP), is widely used for the detection of H_2_O_2_ due to its catalysis capability. The addition of a chromogenic substrate, such as 3,3′,5,5′-tetramethylbenzidine (TMB), is able to produce a color change in the presence of H_2_O_2_.^1,2^ Furthermore, glucose in serum could be oxidized to produce H_2_O_2_ under the catalysis of glucose oxidase (GOx). This produced H_2_O_2_ then reacts with HRP, which could be used to diagnose diabetes.^3–5^ However, high costs of preparation, purification and easy inactivation of HRP motivate people to seek alternative mimic enzymes as complementary peroxidase enzyme to sense glucose for clinical diagnosis.^6–8^ Many nanomaterials exhibit good peroxidase properties, such as Fe_3_O_4_,^9^ Co_3_O_4_,^10^ and Au NPs,^11^ Prussian blue,^12^ carbon nanodots,^13^*etc.* Comparing to HRP, these peroxidase mimetics have advantages of lower cost, flexibility in design, and good chemical stability.

Molybdenum disulfide (MoS_2_) is a typical layered transition metal dichalcogenides (TMDs) formed by a stack of S–Mo–S through weak van der Waals force.^14,15^ MoS_2_ has been regarded as one of the most promising materials due to its unique structure and electronic properties.^16,17^ An intrinsic peroxidase-like activity possessed by MoS_2_ nanomaterials has been densely studied in the past few years.^18–23^ However, most of these MoS_2_ nanomaterials were synthesized by hydrothermal method,^19–21^ which required high temperature and pressure. A few MoS_2_ nanomaterials were prepared by liquid exfoliation,^18,22,23^ but the organic solvent and surfactant were difficult to remove and the residual could affect the peroxidase performance. Zhou *et al.* reported that an alternative way to synthesis MoS_2_ nanosheet,^24^ which could avoid the drawbacks inhered from the above-mentioned methods. Jia *et al.* modified the method introduced by Zhou and successfully fabricated MoS_2_ nanoplates.^25^

Herein, a mixed-solvent liquid exfoliation method on the basis of Jia's has been introduced to synthesis MoS_2_ NPs. A custom made spectrometer has been established to systematically investigate the catalysis performance of MoS_2_ NPs to get the optimized parameters. The as-prepared MoS_2_ NPs exhibits good catalysis capability and the detection limit of H_2_O_2_ and glucose performed by this method could approach 1.25 μM and 7 μM respectively. A test of serum has been conducted by using this method and the results are comparable to that from commercial glucometer. This indicates the practicality of this prepared MoS_2_ NPs and its relative test method could be used as an alternative way for the diagnosis of diabetes. However, there is still much room for improvement, compared with these using high-precision glucometer. More fundamental insights could be provided to improve this flexible and economic method for clinical diagnosis.

## Experimental section

### Chemicals and materials

H_2_O_2_, sodium acetate and acetic acid were obtained from Chongqing Chemical Reagent Company (China). Horseradish peroxidase (HRP, >150 U mg^−1^), glucose, GOx, lactose, sucrose and fructose were obtained from Shanghai Sangon Biotech Co., Ltd (China). 3,3′,5,5′-Tetramethylbenzidine (TMB) were purchased from Merck & Co., Inc (USA). MoS_2_ powder (≥98%, 2 μm in size) were purchased from Tianjin Chemical Reagent Factory Kaida chemical plant. All chemicals and reagents were of analytical grade and used as received without further purification, and ultrapure water was used throughout the work. Ultrapure water was prepared in the lab using a water treatment device. Serum was kindly donated from the Department of Clinical Laboratory at the University-Town Hospital of Chongqing Medical University (Shapingba District, Chongqing Municipality).

### Apparatus

High-resolution transmission electron microscopy (HRTEM) images were carried out with an FEI Tecnai G2 F30 transmission electron microscope (USA). X-Ray photoelectron spectroscopy (XPS) data were recorded in a Thermo Fisher ESCALAB 250xi (England) using ALKα radiation (1486.6 eV). Binding energies were calculated with respect to C (1s) at 284.8 eV. Binding energies were measured with a precision of ±0.05 eV. X-Ray diffraction (XRD) spectra were obtained through a X′-Pert3 Powder X-ray diffractometer (PANalytical, Netherlands). Zeta potential was measured by Zetasizer Nano-ZS (Malvern, UK). The atomic force microscopy (AFM) imagines were performed on a Multi-mode Nanoscope III3d scanning probe microscopy system (Burker, USA). The pH of the solutions was detected by a PHS-3D pH meter (Shanghai Precision Scientific Instruments Co., Ltd., China).

**Fig. 1:**
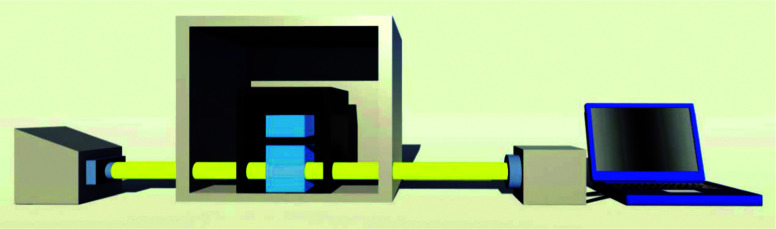
Schematic diagram of this custom made thermal controlled spectrometer. From left to right: tungsten halogen lamp, drying oven, cuvette, spectrometer and computer.

The custom made thermal controlled spectrometer is shown in Fig. 1. Drying oven is used to control the temperature of the cuvette's surrounding environment. Unpolarized light from a tungsten halogen lamp (HL-2000-HP, Ocean Optics) illuminates the cuvette and the transmitted light is collected by spectrometer (HR4000, Ocean Optics). The illumination and absorption of cuvette is conducted through fibers, which dramatically enhance the flexibility of this setup. The spectral information was recorded and analyzed by a program written in C++ and Matlab.

### Preparation of MoS_2_ NPs

The MoS_2_ NPs were prepared according to the mixed-solvent liquid exfoliation method reported previously.^25^ Briefly, 300 mg MoS_2_ powder was added into a 250 mL beaker, 100 mL of ethanol/water with ethanol volume fraction of 45% was added as dispersion solvent. The sealed beaker containing the above mixture was ultrasonicated for 24 h. In order to remove the aggregates, the obtained dark green suspension was centrifuged at 6000 rpm for 20 min three times. After that, collected supernatant was heated at 70 °C in a drying oven to remove the ethanol and water successively. Then the product was resolved in water and centrifuged for 20 min twice at 6000 rpm. At last, the supernatant was further purified by a 0.22 μm Millipore membrane filter. The final product was stored at 4 °C until required for further use.

### Detection of H_2_O_2_ and glucose

The detection of H_2_O_2_ was performed as follows: 0.3 mL of TMB solution (0.8 mM in ethanol) performed as peroxidase substrate and 0.3 mL H_2_O_2_ with different concentrations were added into 2.1 mL of acetate buffer solution (0.2 M, pH = 3.5) in a 4 mL cuvette. Then, 0.3 mL of MoS_2_ NPs solution (100 μg mL^−1^) was injected to the above mixture immediately, and the mixture was incubated at 30 °C for 20 min. The absorption of the mixture was recorded at 656 nm, which is a typical absorption wavelength of oxidized TMB (oxTMB).

The detection of glucose was realized as follows: 0.03 mL of 5.0 mg mL^−1^ glucose oxidase (GOx) and 0.27 mL of glucose with different concentrations in 10 mM acetate buffer solution (pH 5.5) were incubated at 37 °C for 30 min to produce H_2_O_2_. The other procedure of detection was the same as that of H_2_O_2_.

In selectivity experiment, the target was replaced by sucrose (1.35 mM), lactose (1.35 mM) and fructose (1.35 mM) respectively during the experiment while the detection procedure was the same as that of glucose.

For the detection of glucose in human serum, the proteins in serum samples were separated by modified precipitation process according to the literature.^18^ Firstly, diluting 0.3 mL of serum with 0.2 mL water, then adding 0.5 mL Ba(OH)_2_ (0.11 M) and 0.5 mL ZnSO_4_ (0.0765 M) to above mixture. After centrifugation at speed of 4000 rpm for 15 min, 0.5 mL of the supernatant was extracted and diluted with 1.5 mL of acetate buffer solution (10 mM, pH 5.5). The other detection process was the same as the above mentioned glucose detection procedure.

## Results and discussion

### Characterization of MoS_2_ NPs

**Fig. 2:**
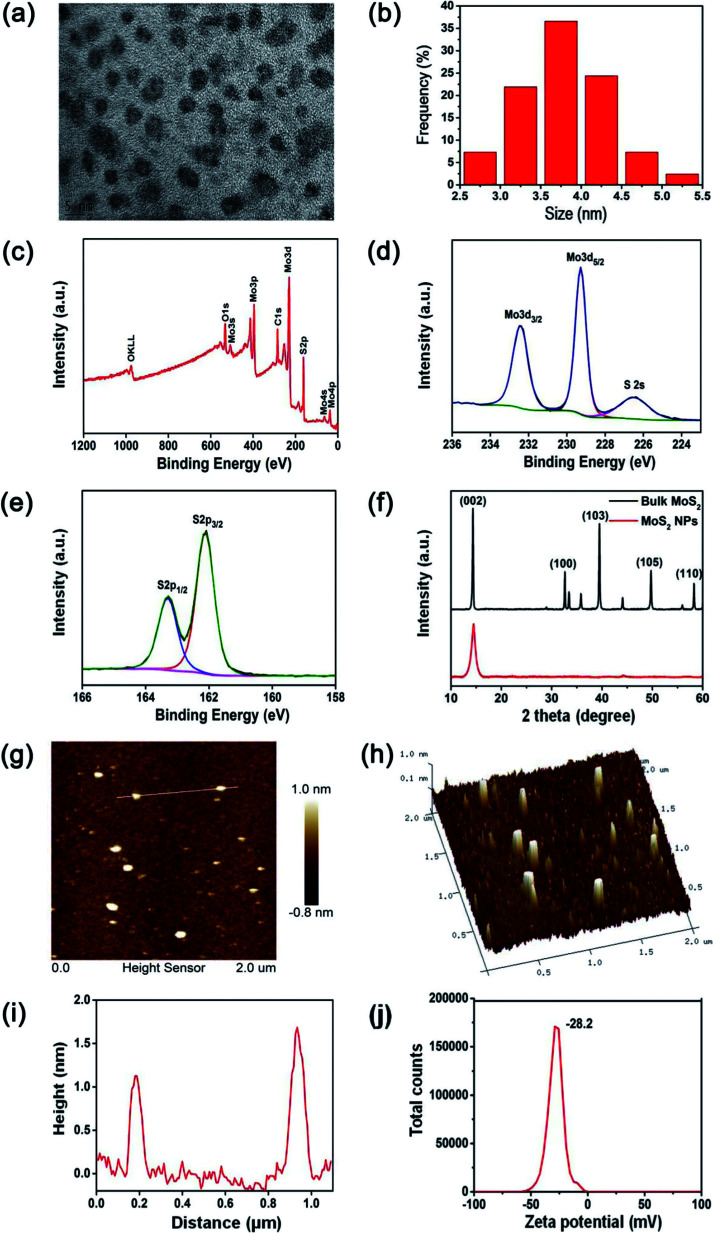
(a) HRTEM image of MoS_2_ NPs, (b) size distribution of MoS_2_ NPs, (c) XPS spectrum of MoS_2_ NPs, (d) high-resolution peak-fitting spectra of the Mo 3d, (e) high-resolution peak-fitting spectra of the S 2p, (f) XRD patterns of bulk MoS_2_ crystals and MoS_2_ NPs, (g) AFM image of MoS_2_ NPs, (h) AFM 3D height profile of MoS_2_ NPs, (i) height profile along the white line shown in the AFM image, (j) zeta potential distribution of MoS_2_ NPs.

MoS_2_ NPs were obtained from the bulk MoS_2_ by the simple mixed-solvent liquid exfoliation method. The high resolution transmission electron microscopy (HRTEM) image of the resulting MoS_2_ NPs shown in Fig. 2(a) indicates that these NPs are spherical and well-dispersed, with an average diameter of 3.7 nm (Fig. 2(b)). The XPS spectrum shown in Fig. 2(c) demonstrates the Mo and S elemental peaks. And the high-resolution XPS spectra were collected to analyze the chemical states of Mo and S in MoS_2_ NPs. The peaks at 232.4 and 229.3 eV observed by the high-resolution spectrum of Mo (Fig. 2(d)) are attributed to Mo^4+^ 3d_3/2_ and Mo^4+^ 3d_5/2_, respectively. In addition, the peaks and 163.3 and 162.1 eV shown in the high-resolution spectrum of S (Fig. 2(e)) are corresponding to the S 2p_1/2_ and S 2p_3/2_ orbits of divalent sulfide ions (S^2−^).^22,26^ XRD was used to further investigate the crystal structure of the prepared MoS_2_ NPs and bulk MoS_2_ crystals. As shown in Fig. 2(f), both bulk MoS_2_ crystals and MoS_2_ NPs are mainly identified as 2H-MoS_2_, which has a dominant peak at 14.3°, representing the (002) plane (JCPDS 37-1492). In addition, the broadening (002) peak and the absence of most other peaks of MoS_2_ NPs provide a direct evidence of a decrease in the particle size.^22,23^ The thickness of the MoS_2_ NPs is around 1–2 nm, as shown in the atomic force microscopy (AFM) image and height profile of MoS_2_ NPs (Fig. 2(g)–(i)). The zeta potential of the MoS_2_ NPs was measured to be −28.2 mV (Fig. 2(j)). This indicates that MoS_2_ NPs exhibit a great colloidal stability in aqueous media.

### Peroxidase-like activities of MoS_2_ NPs

The peroxidase-like activity of MoS_2_ NPs was investigated in terms of the catalytic oxidation of the peroxidase substrate TMB in the presence of H_2_O_2_ under acidic conditions (pH = 4). According to previous literatures,^18,20,26^ the reaction could be characterized as the following process:

Total: 2H^+^ + TMB + H_2_O_2_ → oxTMB^2+^ + 2H_2_O

(a) H_2_O_2_ → 2OH

(b) TMB + 2OH → oxTMB^2+^ + 2OH^−^

(c) 2H^+^ + 2OH^−^ → 2H_2_O

**Fig. 3:**
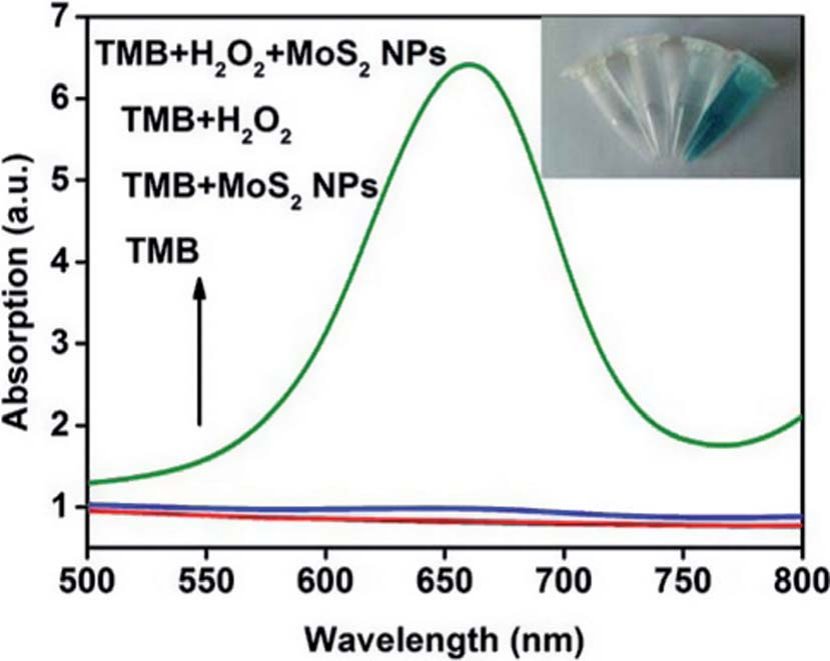
Absorption spectra changes at 656 nm corresponding to (from bottom to top): TMB, TMB–MoS_2_ NPs, TMB–H_2_O_2_ and TMB–H_2_O_2_–MoS_2_ NPs in pH 4.0 acetate buffer (0.2 M) at 40 °C for 10 min. Inset: images of different systems (from left to right): TMB, TMB–MoS_2_ NPs, TMB–H_2_O_2_ and TMB–H_2_O_2_–MoS_2_ NPs. The concentration of MoS_2_ NPs, TMB, and H_2_O_2_ are 10 μg mL^−1^, 0.8 mM, 0.08 mM, respectively.

A series of absorption spectra for comparison is shown in Fig. 3. The absorption spectra of TMB and TMB–MoS_2_ NPs systems have no significant difference in the range between 500 nm and 800 nm. Although TMB–H_2_O_2_ system exhibits a little absorption in 656 nm, it could be ignored compared to spectrum collected from TMB–H_2_O_2_–MoS_2_ NPs system, which displays a strong absorption peak at 656 nm. This is a typical peak of the oxidation products of TMB. The inset images represent different systems after 10 min of reaction. TMB and TMB–MoS_2_ NPs systems remained colorless, which means no oxidation reaction occurred. TMB–H_2_O_2_ system shows a pale green color, which is due to the slow oxidation of TMB by the presence of H_2_O_2_. However, the TMB solution turns to be blue after the addition of MoS_2_ NPs and H_2_O_2_. These results suggest that the prepared MoS_2_ NPs have the peroxidase-like catalysis capability and could effectively catalyze the oxidation of TMB by H_2_O_2_. MoS_2_ NPs could facilitate the electron transfer between TMB and H_2_O_2_ in the oxidation of TMB catalyzed by MoS_2_ NPs. In this process, TMB molecules are absorbed on the surface of MoS_2_ NPs, and donate lone-pair electrons from the amino groups to MoS_2_ NPs, resulting in the increase in electron density and mobility in the MoS_2_ NPs. This will accelerate the electron transfer from the MoS_2_ NPs to H_2_O_2_, and promote the decomposition of H_2_O_2_ in acidic media into ·OH, which oxidizes TMB to form a blue product.^18,20^

### Optimization of experimental conditions

The optimization of detection parameters, including the concentration of MoS_2_ NPs, temperature, pH and reaction time were investigated to enhance the catalysis capability of MoS_2_ NPs. Fig. S1(a)† shows the time-dependent absorption changes against the different concentrations of MoS_2_ NPs. The change in absorption approached the maximum when 10 μg mL^−1^ MoS_2_ NPs was utilized. The effect of temperatures on the catalytic activity was shown in Fig. S1(b).† It can be found that MoS_2_ NPs show good activity over a wide temperature range from 25 °C to 45 °C, and 30 °C was chosen as the optimal temperature for the subsequent experiments. In addition, the catalytic activity was also pH-dependent (Fig. S1(c)†) and the buffer solution with pH of 3.5 was employed for the further experiments. Finally, the effect of reaction time on the catalytic activity of MoS_2_ NPs was investigated, which was shown in Fig. S1(d).† Obviously, 20 min was the optimal reaction time because the maximum change in the spectra could be achieved and the absorption had little change from this time. Therefore, the optimal MoS_2_ NPs concentration, temperature, pH, and reaction time were selected which are 10 μg mL^−1^, 30 °C, 3.5, and 20 min, respectively, and they are used in the following experiments.

### Kinetic assay of peroxidase-like activity of MoS_2_ NPs

The peroxidase-like catalytic behavior of MoS_2_ NPs was studied by employing the steady-state kinetics. The kinetic parameters were harvested through altering the concentration of H_2_O_2_ or TMB while holding the other one invariable. The adsorption of MoS_2_ NPs monitored at the wavelength of 656 nm was recorded from 0–60 s during the peroxidase-like activity. The transient of adsorption can be fitted to the equation, *I* = *vt* + *b*, where *v* is the velocity characterized the adsorption change rate between the initial and final stage, *b* is the initial value of adsorption and *t* is the time. The maximum initial velocity (*V*_max_) and Michaelis–Menten constant (*K*_m_) shown in Table S1† were calculated using the Lineweaver–Burk plot, 1/*v* = (*K*_m_/*V*_max_) × (1/*S*) + 1/*V*_max_, where *v* is the initial velocity and *S* is the concentration of the substrate.^27–30^*K*_m_ usually indicates the affinity of the enzyme to the substrate. The smaller the *K*_m_ is, the stronger the affinity will be.^20,31,32^

**Fig. 4:**
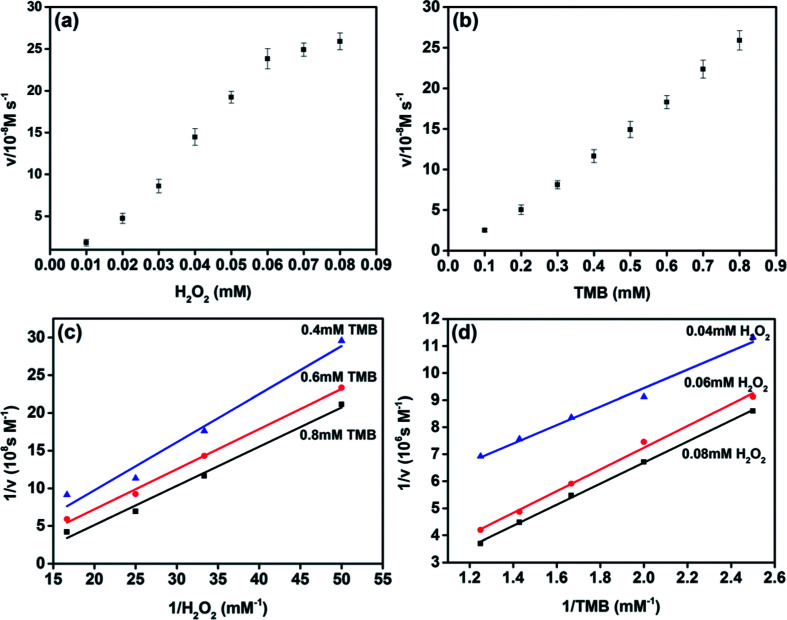
Steady-state kinetic assay and catalytic mechanism of MoS_2_ NPs (a–d). The concentration of MoS_2_ NPs was fixed at 10 μg mL^−1^ in 3 mL of acetate buffer solution (0.2 M, pH = 3.5) at 30 °C. (a) The concentration of TMB was 0.8 mM and the H_2_O_2_ concentration was varied. (b) The concentration of H_2_O_2_ was 0.08 mM and the TMB concentration was varied. (c) and (d) Double-reciprocal plots of activity of MoS_2_ NPs at a fixed concentration of one substrate and the other varied. The error bars represent the standard error derived from three repeated measurements.

The apparent *K*_m_ value of MoS_2_ NPs with H_2_O_2_ as substrate was obviously lower than that of HRP, which indicates that MoS_2_ NPs exhibits higher affinity to H_2_O_2_ than that of HRP. The apparent *K*_m_ value of MoS_2_ NPs with TMB as substrate was higher than that of HRP, is in accordance with the observation that a higher concentration of TMB was required to utilize to achieve the optimal activity of MoS_2_ NPs. To further investigate the catalysis mechanism of MoS_2_ NPs, the activity employing a wide range of TMB and H_2_O_2_ concentrations was carried out. The double-reciprocal plots of initial velocity *versus* different concentrations of one substrate were acquired while the concentration of this counterpart is set to be invariable (Fig. 4). The parallel slope of the lines reveals a ping-pong mechanism.^18,20,33^ In other words, the MoS_2_ NPs bond and react with the first substrate, and then release the first product before reacting with the second substrate.

### Detection of H_2_O_2_ and glucose

**Fig. 5:**
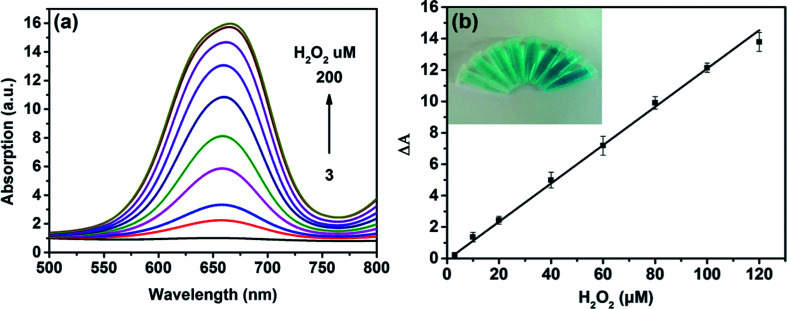
(a) Absorption spectra changes in the presence of different H_2_O_2_ concentrations (intensity from weak to strong: 3, 10, 20, 40, 60, 80, 100, 120, 160, 200 μM). (b) The linear calibration plot for H_2_O_2_ (3, 10, 20, 40, 60, 80, 100, 120 μM). Δ*A* = Abs (20 min, 656 nm) − Abs (0 min, 656 nm). The inset of (b) are the images of color changes for the corresponding concentrations of H_2_O_2_.

On the basis of the intrinsic peroxidase-like property of MoS_2_ NPs, a colorimetric approach for the detection of H_2_O_2_ and glucose was utilized under the optimal experimental conditions. Fig. 5(a) shows the absorption spectra of MoS_2_ NPs with different H_2_O_2_ concentration. It can be seen that the catalytic activity of MoS_2_ NPs is dependent on the concentration of H_2_O_2_. Fig. 5(b) exhibits the calibration plot for H_2_O_2_. It shows that the absorption of the mixture has a good linear relationship with the concentration of H_2_O_2_ ranging from 3 to 120 μM (*R*^2^ = 0.9983) and the detection limit is calculated to be 1.25 μM. A comparison of this approach with other colorimetric methods for H_2_O_2_ detection is listed in Table S2.† It is shown that by employing MoS_2_ NPs produced by this method, this sensing ability to H_2_O_2_ is comparable to other colorimetric methods.

**Fig. 6:**
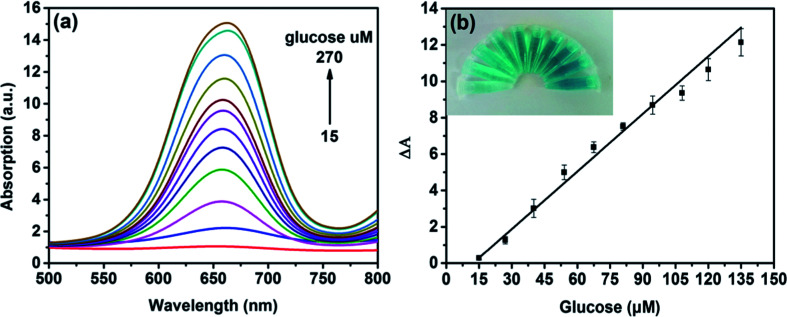
(a) Absorption spectra changes in the presence of different glucose concentrations (intensity from weak to strong: 15, 27 40.5, 54, 67.5, 81, 94.5, 108, 121.5, 135, 189, 270 μM). (b) The linear calibration plot for glucose detection (15, 27 40.5, 54, 67.5, 81, 94.5, 108, 121.5, 135 μM). The inset of (b) are the images of color changes for the corresponding concentrations of glucose.

Since H_2_O_2_ is the main product during the GOx-catalyzed reaction, this sensing strategy can be employed for the determination of glucose when MoS_2_ NPs is combined with glucose oxidase (GOx). GOx can catalyze glucose oxidation to generate H_2_O_2_ and the following produced H_2_O_2_ can further oxidize TMB to bring a blue-color product through the catalysis of MoS_2_ NPs. Fig. 6(a) illustrates the absorption spectra of different concentrations of glucose from 15 to 270 μM. Fig. 6(b) shows the calibration curve for detecting glucose with a linear range from 15 to 135 μM. Moreover, the detection limit was found to be as low as 7 μM. Also, the LOD obtained by this method was much lower than the level of about 1 mM for glucose in human blood serum, indicating that the probe was suitable for detection of glucose in clinic.

The recovery was performed and the results are shown in Tables S4 and S5.† It can be seen that the recoveries are range from 95% to 99%. These results showed that this proposed method has a promising reliability for the detection of H_2_O_2_ and glucose.

### Selectivity analysis for glucose detection

NaCl, KCl, NH_4_Cl *etc.* are utilized to test the selectivity of the MoS_2_ NPs-based sensing method towards H_2_O_2_. Mixture of 40 μM H_2_O_2_ and various concentrations of these impurities were measured and the results are shown in Table S6.† The negligible change in absorption of these mixtures compared to that collected from pure H_2_O_2_ indicates the presence of these impurities has little effect on the detection of H_2_O_2_.

**Fig. 7:**
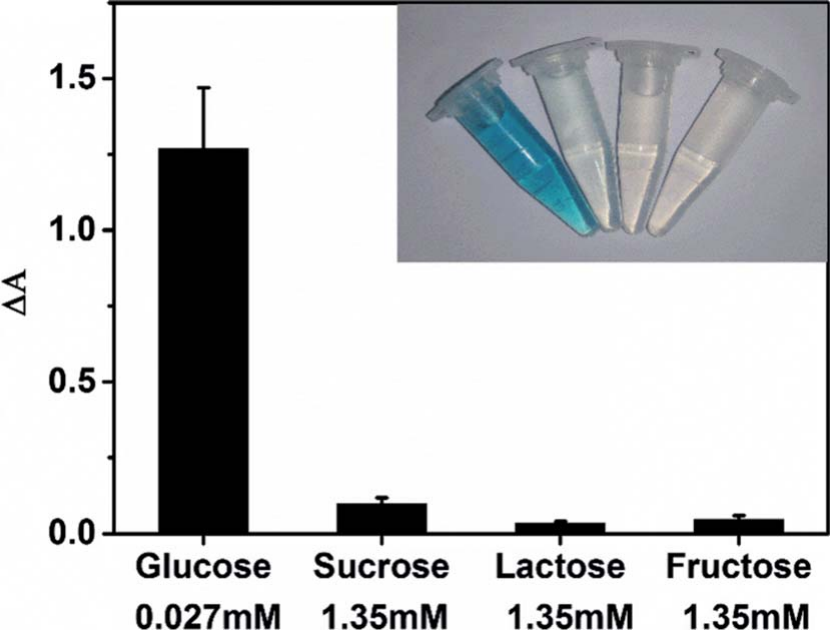
Selectivity analysis for glucose detection using other sugars as control (the concentration was 0.027 mM for glucose, 1.35 mM for sucrose, 1.35 mM for lactose, 1.35 mM for fructose.). Inset of this figure were images of colored production corresponding to the above four solutions. The error bars represent the standard deviation of three measurements.

The selectivity of MoS_2_ NPs-based sensing method towards glucose was investigated in a series of control experiments. Some alternative sugars including sucrose, lactose and fructose were examined using the same detection procedure as that of glucose. Fig. 7 shows that no obvious interference is observed even the concentrations of other sugars are 50 times higher than that of glucose. Therefore, this MoS_2_ NPs produced here has a high selectivity for glucose detection because of the high specificity of GOx towards glucose.

### Serum analysis

**Fig. 8:**
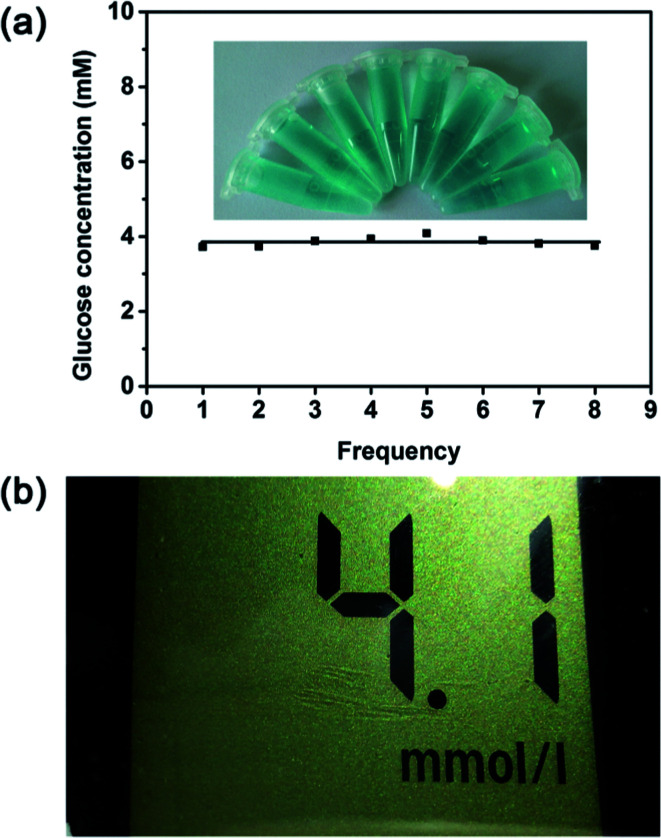
(a) Reproducibility experiments of the colorimetric detection of glucose in serum samples. Inset of (a) are the corresponding color change of each independent experiment. (b) The glucose concentration in serum sample tested through a medical electronic glucometer.

The glucose detection strategy reported here was then employed to detect the real samples to prove its practicability. Serums were selected as the detecting targets. On the basis of this method described in previous section, the glucose level in the human serum sample was tested. As shown in Fig. 8a, the glucose concentration of the serum sample is calculated to be 3.85 mM, which is not much different from the result obtained from the medical electronic glucometer (4.1 mM) (Fig. 8b). To confirm its suitability for real assay, another three different serum samples were used for comparison. As illustrated in Table S7,† the detection results of proposed method agree well with the detection results of glucometer. Therefore, this proves that this method initiated here is applicable for the glucose detection in human serum.

The serum used in this paper was kindly donated from the Department of Clinical Laboratory at the University Town Hospital of Chongqing Medical University (Shapingba District, Chongqing Municipality) with the permission of the patients.

This donation and experiment conducted in study was in compliance with the regulations issued by the Chongqing Medical University.

## Conclusions

MoS_2_ NPs in ultra-small size was synthesized by a simple facile liquid exfoliation method in the presence of ethanol/water mixture. This environmental friendly and economic fabrication method benefits from the utilization of ethanol/water mixture. A custom-made thermal controlled spectrometer has been established to analyze the intrinsic peroxidase-like activity of the prepared MoS_2_ NPs. The detection of H_2_O_2_ and glucose with high sensitivity and good selectivity by using this MoS_2_ NPs has been demonstrated and the MoS_2_ NPs exhibits a good performance to sense the serum. The potential of MoS_2_ NPs used as catalyst for other oxidation reactions could be carried out in the future research and this could create a new opportunity for this enzyme-mimicking MoS_2_ NPs in various significant fields.

## Conflicts of interest

There are no conflicts to declare.

## Supplementary Material

RA-008-C7RA12584D-s001
