# Supplementary material for: Mixed-solvent liquid exfoliated MoS2 NPs as peroxidase mimetics for colorimetric detection of H2O2 and glucose
Source: RSC Adv. 2018 Feb 15;8(13):7252–9. doi: 10.1039/c7ra12584d (PMC9078403; doi:10.1039/c7ra12584d)
Supplement: RA-008-C7RA12584D-s001 [file RA-008-C7RA12584D-s001.pdf]

Electronic Supplementary Material (ESI) for RSC Advances.
This journal is © The Royal Society of Chemistry 2018

Electric Supplenmentary Material

A mixed-solvent Liquid exfoliated MoS_2 nanoparticles as peroxidase mimetics for colorimetric detection of $H_2O_2$and glucose

Yuehua Zhao[a,b,#], Yu Huang[a,#,*], Jiangling Wu[c], Xiaoli Zhan[d], Yuanyang Xie[a,b], Dongyun Tang[a], Haiyan Cao[e,*], Wen Yun[f,*]

[a] *Chongqing Institute of Green and Intelligent Technology, Chinese Academy of Sciences, Chongqing, 400714, China*

[b] *University of Chinese Academy of Science, Beijing, 100049, China*

[c] *University-Town Hospital of Chongqing Medical University, Chongqing , 401331, China*

[d] *Chongqing Traditional Chinese Medicine Hospital, Chongqing, 400021, China*

[e] *School of Chemistry and Chemical Engineering, Yangtze Normal University, 408100,Chongqing, China*

[f] *Chongqing Key Laboratory of Catalysis and Functional Organic Molecules, College of Environment and Resources, Chongqing Technology and Business University, Chongqing, 400067, China*

# These authors contributed equally to this work

*Corresponding author email address: huangyu@cigit.ac.cn (Yu Huang), 513923170@qq.com (Haiyan Cao),yunwenyunwen@126.com (Wen Yun)

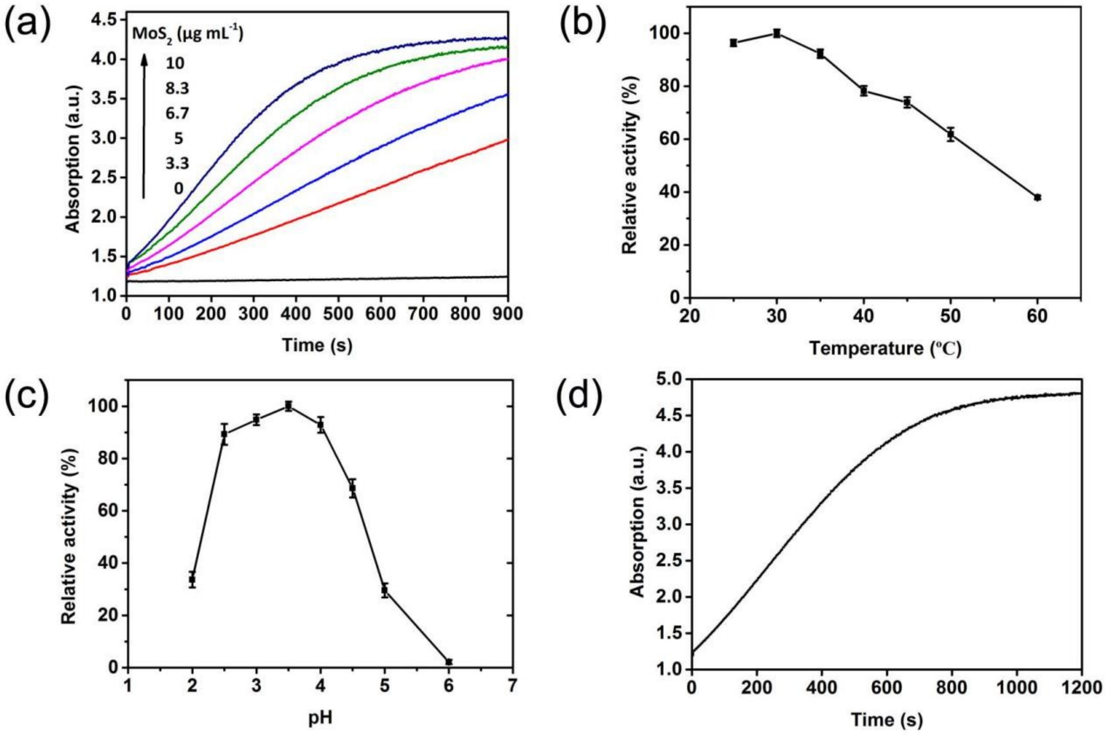

Fig.S1 (a) The time-dependent absorption changes at 656 nm in the presence of different concentrations of MoS_2 NPs in HAc-NaAc buffer (pH 4.0) at 40°C with 0.8 mM TMB and 0.04 mM H_2O_2 for 15 min .

Effect of temperature (Fig.S1(b)) and pH (Fig.S1(c)) on the catalytic activity of MoS_2 NPs.

Reaction condition of (b) : 300 μL MoS_2 NPs (100 μg mL^{-1}) + 300 μL TMB(8 mM)+ 300 μL H_2O_2 (0.4 mM) + 2100 μL HAc-NaAc buffer (pH 4.0).

Reaction condition of (c) : 300μL MoS_2 NPs (100 μg mL^{-1}) + 300 μL TMB(8 mM)+ 300 μL H_2O_2 (0.4 mM) + 2100 μL HAc-NaAc buffer (different pH values).

The error bars represent the standard deviation of three measurements.

Fig.S1(d) The effect of reaction time on the catalytic activity of MoS_2 NPs. The experiment was carried out using 10ug mL^{-1} MoS_2 NPs in a reaction volume of 3 mL, in HAc-NaAc buffer (pH 3.5) with 0.8 mM TMB and 0.04 mM H_2O_2 for 20 min at 30℃.

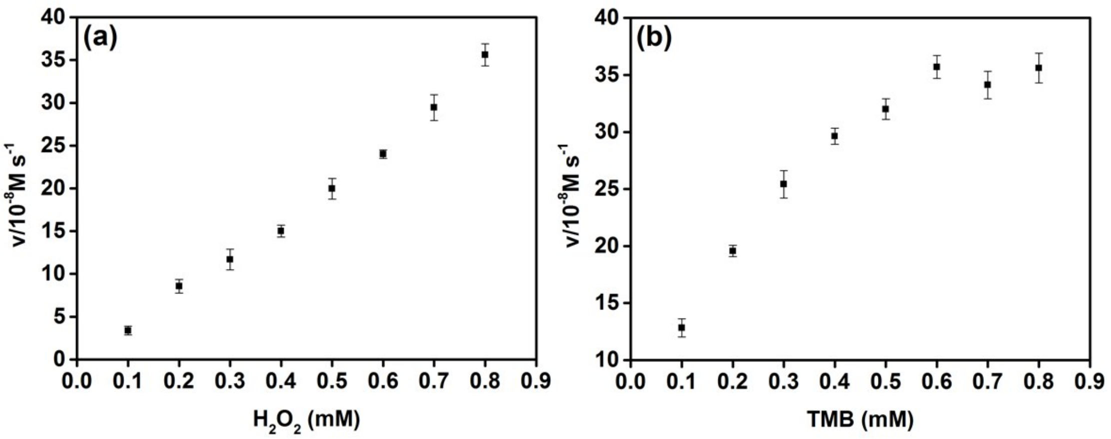

Fig.S2 Steady-state kinetic assay of HRP. The velocity (*v*) of the reaction was measured using HRP (5 μg L^{-1}) in 3 mL of acetate buffer solution (0.2M, pH = 3.5) at 30 °C. (a) The concentration of TMB was 0.8mM and the H_2O_2 concentration was varied. (b) The concentration of H_2O_2 was 0.8mMand the TMB concentration was varied.

Table.S1 Comparison of the kinetic parameters of MoS_2 NPs and HRP. K_m is the Michaelies constant, V_{max} is the maximal reaction velocity.

Catalyst	Substrate	K_m (mM)	V_{max} (10^{-8}M s^{-1})
MoS_2 NPs	H_2O_2	0.1355	28.4
MoS_2 NPs	TMB	2.5324	58.9
HRP	H_2O_2	2.5547	85.5
HRP	TMB	0.2934	50.1

Table.S2 Comparison of various colorimetric methods for detection of H_2O_2.

Materials	LOD(μM)	Linear range(M)	Reference
Fe_3O_4 MNPs	3	5×10^{-6} -1×10^{-4}	1
Positively charged AuNPs	0.5	2×10^{-6} -2×10^{-4}	2
SDS- MoS_2 NPs	0.32	2×10^{-6} -1×10^{-4}	3
MoS_2/PPy Nanocomposite	45	50×10^{-6} -2×10^{-3}	4
MoS_2 nanosheets	1.5	5×10^{-6} -1×10^{-4}	5
MoS_2 NPs	1.25	3×10^{-6} -1.2×10^{-4}	This work

Table.S3 Comparison of various colorimetric methods for detection of glucose.

Materials	LOD(μM)	Linear range(M)	Reference
Fe_3O_4 MNPs	30	50×10^{-6} -10×10^{-4}	1
Positively charged AuNPs	4	18×10^{-6} -11×10^{-4}	2
SDS - MoS_2 NPs	0.57	5×10^{-6} -5×10^{-4}	3
PVP - MoS_2 NPs	320	1000×10^{-6}-100×10^{-4}	6
MoS_2 nanosheets	1.2	5×10^{-6} -1.5×10^{-4}	5
MoS_2 NPs	7	15×10^{-6} -1.35×10^{-4}	This work

Table.S4 Detection results of H_2O_2 after adding different concentrations of H_2O_2 into known concentration of H_2O_2

Sample number	Content (μM)	Added (μM)	Measured (μM)	Recovery (%,n=3)	RSD (%,n=3)
1	5	5	9.83	98.3	4.8
2	5	35	38.76	96.9	6.3
3	5	75	77.21	96.5	5.6

Table.S5 Detection results of glucose after adding different concentrations of glucose into known concentration of glucose

Sample number	Content (μM)	Added (μM)	Measured (μM)	Recovery (%,n=3)	RSD (%,n=3)
1	10	10	19.12	95.6	5.3
2	10	50	58.68	97.8	6.7
3	10	90	97.1	97.1	6.4

Table.S6 Influence of foreign substances on the detection of 40 μM H_2O_2 using the proposed method.

Foreign substance	Concentration (mM)	Change in absorption signal (%)[n]
NaCl	500	+5.2
KCl	500	+4.9
NH_4Cl	500	+3.1
$CaCl_2$	100	+3.8
$MgCl_2$	100	+3.3
$BaCl_2$	100	+4.4
$NiCl_2$	100	+4.9
Glucose	20	-5.5
Tryptophan	5	-4.6
NaClO	0.1	+5.9

[n]The average value of five experiments

Table.S7 Results of determination of glucose in human serum samples (n=3).

Sample number	Glucometer method (mM)	Proposed method (mM)	RSD (%)
1	5.6	5.17 ± 0.06 [n]	-7.7%
2	6.4	6.03 ± 0.05 [n]	-5.8%
3	7.7	7.16 ± 0.05 [n]	-7.0%

References

1. H. Wei and E. Wang, *Anal. Chem.*, 2008, **80**, 2250.
2. Y. Jv, B. Li and R. Cao, *Chem. Commun.*, 2010, **46**, 8017.
3. K. Zhao, W. Gu, S. Zheng, C. Zhang and Y. Xian, *Talanta*, 2015, **141**, 47.
4. J. Lei, X. Lu, G. Nie, Z. Jiang and C. Wang, *Part. Part. Syst. Char.*, 2015, **32**, 886.
5. T. Lin, L. Zhong, L. Guo, F. Fu and G. Chen, *Nanoscale*, 2014, **6**, 11856.
6. J. Yu, X. Ma, W. Yin and Z. Gu, *RSC Adv.*, 2016, **6**, 81174.
